# Laparoscopic Management of Long-Standing Gallbladder Hydrops: A Case Report and Literature Review

**DOI:** 10.7759/cureus.92744

**Published:** 2025-09-19

**Authors:** Ahmed R Zubi, Imran M Adam, Ibrahim S Abdulla, Hawwa Nuha, Abdulla Ubaid

**Affiliations:** 1 General Surgery, Indira Gandhi Memorial Hospital, Male, MDV

**Keywords:** cholecystectomy, cystic duct obstruction, gallbladder hydrops, gallbladder mucocele, impacted stone, intraoperative decompression

## Abstract

Gallbladder hydrops, a condition characterized by distension of the gallbladder with clear serous fluid due to chronic outflow obstruction, is an uncommon clinical entity most often associated with cholelithiasis. It poses a diagnostic and therapeutic challenge, as it can present with a wide spectrum of symptoms, from being an incidental finding to mimicking other abdominal pathologies. This case report illustrates the clinical course of a patient with long-standing, intermittently symptomatic gallbladder hydrops.

A 63-year-old woman presented with a four-year history of intermittent right upper quadrant pain. Imaging three years prior (ultrasound and CT) had confirmed the diagnosis of gallbladder hydrops with an impacted stone in the cystic duct. She deferred surgery at that time. Upon re-presentation, imaging revealed a persistently distended gallbladder. She successfully underwent a laparoscopic cholecystectomy, which required intraoperative percutaneous decompression to aspirate 125 mL of clear fluid and facilitate safe dissection. The procedure was uncomplicated, and the patient was discharged on postoperative day one. Histopathology confirmed chronic cholecystitis.

This case highlights that gallbladder hydrops can have a prolonged and indolent symptomatic course. Laparoscopic cholecystectomy remains the definitive treatment but can be technically demanding due to the distended, tense gallbladder. Preoperative recognition and preparedness for intraoperative decompression are crucial techniques for minimizing the risk of iatrogenic perforation and ensuring a successful surgical outcome. A review of the literature underscores the variable presentation and management strategies for this condition.

## Introduction

Gallbladder hydrops is a benign condition resulting from a prolonged blockage of the gallbladder outflow, which leads to its filling with clear serous fluid. It most commonly occurs due to an impacted stone in the gallbladder neck or cystic duct [1]. Other possible causes include cystic duct fibrosis, tumors (of the gallbladder or cystic duct), certain parasitic infections, external compression, and motility disorder [2]. With prolonged obstruction, the bile salts are absorbed, and the bile is replaced with a clear serous fluid. The gallbladder distends to various degrees, and volumes of up to 1500 ml have been reported [3].

As gallbladder hydrops is primarily a complication of cholelithiasis, it occurs most commonly in middle-aged women [3]. It usually follows an episode of acute cholecystitis, which may resolve, but the blockage of the cystic duct persists, resulting in chronic intermittent pain and bloating [4]. In some instances, it may be discovered incidentally [5]. It can also present with unusual symptoms such as lower abdominal pain, urinary frequency, gastric outlet obstruction, or Mirizzi’s syndrome [1,4,6,7]. More serious complications, such as empyema or perforation, may also occur [8].

Laboratory results are often within the reference range for uncomplicated cases. Abdominal ultrasound, computed tomography (CT), or magnetic resonance cholangiopancreatography (MRCP) typically reveal a distended gallbladder, commonly with associated cholelithiasis [9]. Laparoscopic cholecystectomy is the standard treatment for gallbladder hydrops. However, the procedure becomes more technically challenging with an increasing risk of perforation [10]. Percutaneous drainage serves as an alternative for acutely ill patients, acting as a bridge to definitive surgery [6].

We present the case of a middle-aged woman with long-standing gallbladder hydrops who had intermittent symptoms and was treated with laparoscopic cholecystectomy. A comparative review of the literature on its characteristics, clinical features, diagnostic imaging, and treatment is also provided.

## Case presentation

A 63-year-old woman presented to the surgical OPD as a known case of gallbladder hydrops, diagnosed three years prior. For four years, she had occasional episodes of right upper abdominal pain, characterized as mild, dull, radiating to the upper back and suprapubic area. The pain was precipitated by heavy meals or prolonged fasting and was relieved spontaneously or with simple analgesics.

**Figure 1:**
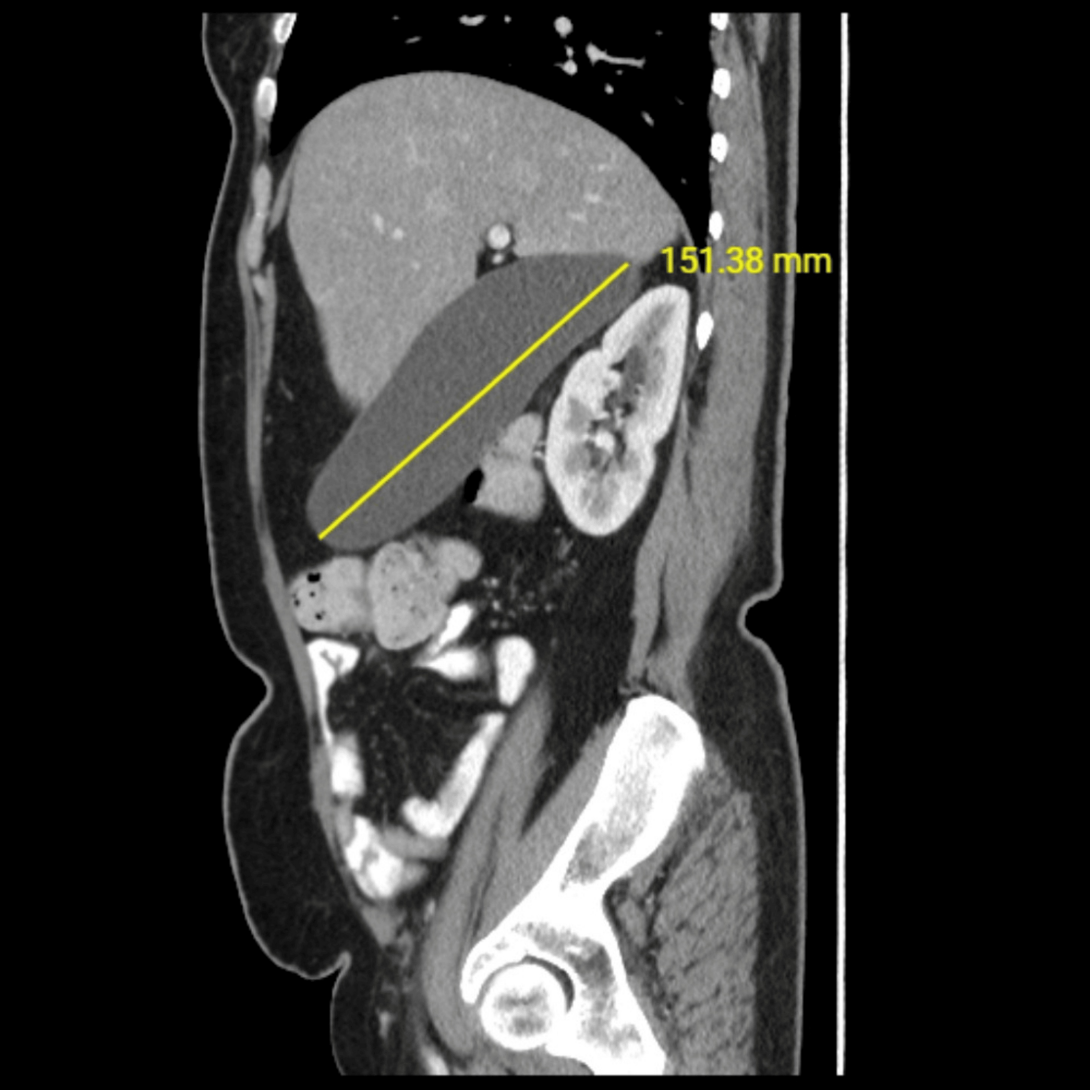
Abdominal CT at the first presentation Abdominal CT from the first presentation (three years before the cholecystectomy detailed in this case report) showing a distended gallbladder.

Three years prior to her presentation, she sought medical advice for urinary symptoms. An abdominal ultrasound revealed a distended gallbladder with multiple stones, including one impacted stone at the neck, with high suspicion of a hydropic gallbladder. This was followed by an abdominal CT, which confirmed the diagnosis (Figure 1). The laboratory investigations were within the reference range (Table 1). She was offered laparoscopic cholecystectomy but deferred the surgery due to social reasons.

**Table 1:** Patient’s laboratory investigations

Parameter	Three years prior to surgery	15 days prior to surgery	Reference Value
Hemoglobin	13.6 g/dL	12.9 g/dL	10.6 - 13.5 g/dL
White cell count	8.14 10^9^/L	9.47 10^9^/L	4.37 - 9.68 10^9^/L
Platelet	237 10^9^/L	257 10^9^/L	186 - 353 10^9^/L
Urea	23.54 mg/dL	25.68 mg/dL	15 - 40 mg/dL
Creatinine	0.82mg/dL	0.81 mg/dL	0.57 - 1.11 mg/dL
Serum sodium	142 mmol/L	140 mmol/L	136 - 145 mmol/L
Serum potassium	4.4 mmol/L	4.3 mmol/L	3.5 - 5.1 mmol/L
Total bilirubin	0.8 mg/dL	0.4 mg/dL	0.2 - 1.2 mg/dL
Albumin	5 g/dL	4.5 g/dL	3.5 - 5.2 g/dL
Alkaline phosphatase	49 U/L	95 U/L	40 - 150 U/L
aspartate aminotransferase	33 U/L	51 U/L	5.0 - 34 U/L
alanine aminotransferase	62 U/L	62 U/L	0 - 55 U/L
Gamma-glutamyl transferase	23 U/L	36 U/L	9.0 - 36 U/L
Fasting Blood Glucose	98 mg/dL	111 mg/dL	60 - 139 mg/dL
Lipase	-	42.78 U/L	8 - 78 U/L

During this three-year period, she experienced mild episodes of pain that resolved spontaneously or with basic analgesics without requiring medical consultation. However, one year prior to presentation, the pain became more frequent and was associated with nausea and anorexia. A systematic review was positive only for chronic constipation. She denied fever, vomiting, dark urine, pale stools, fatigue, or weight loss. She is a known case of diabetes mellitus and dyslipidemia, diagnosed during her initial presentation three years ago, and was started on oral medication. Apart from a cesarean section performed over 20 years ago, she has no history of other surgical interventions and no significant family history.

**Figure 2:**
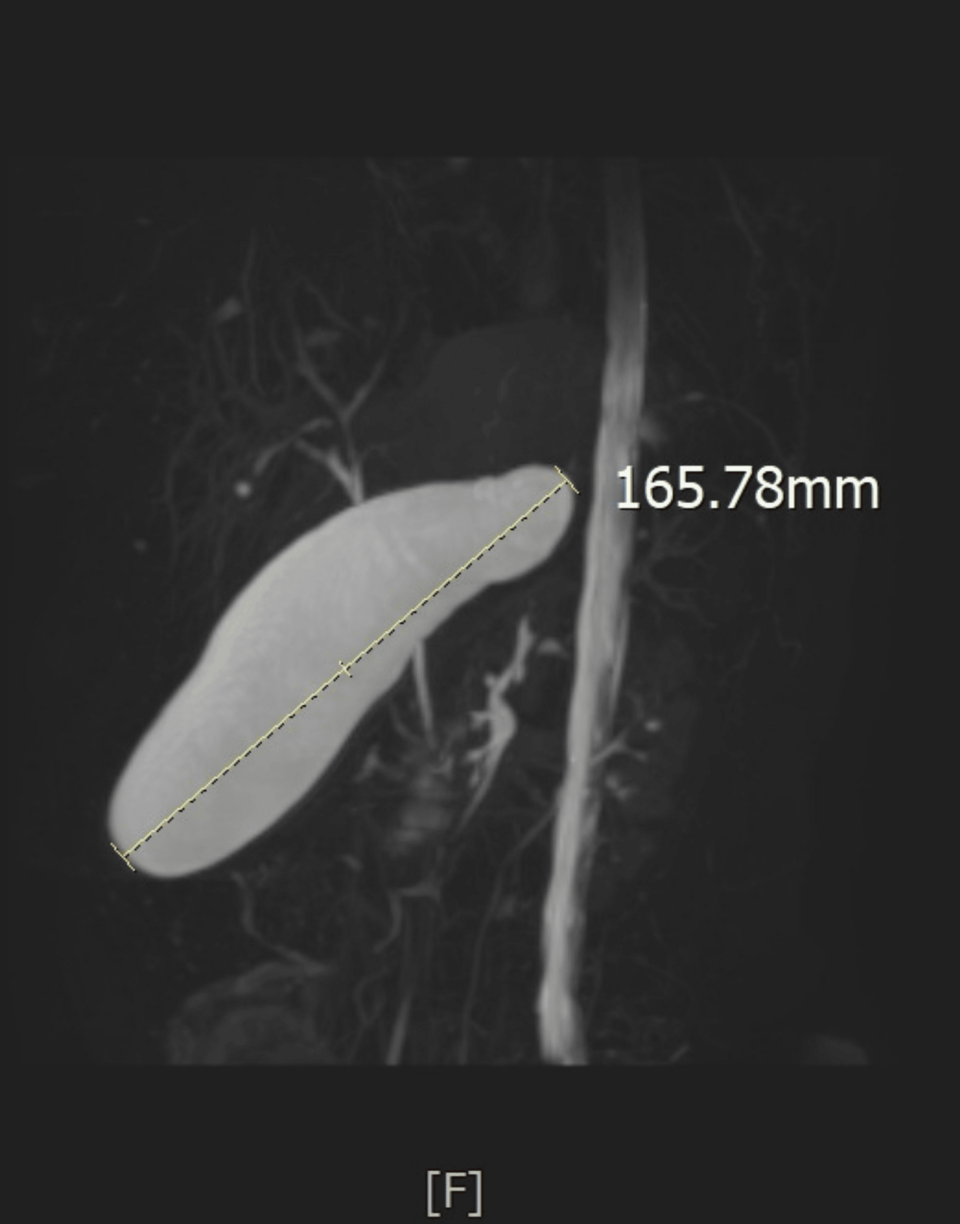
MRCP at second presentation Magnetic resonance cholangiopancreatography (MRCP) from the second presentation (two weeks before the cholecystectomy detailed in this case report) showing a distended gallbladder.

Physical examination revealed an overweight woman who was hemodynamically stable. Her abdomen was flabby, soft, and non-tender, with no palpable gallbladder. Laboratory findings were within normal limits (Table 1). Abdominal ultrasound demonstrated a 13-mm stone impacted in the cystic duct, causing gallbladder dilatation. Magnetic resonance cholangiopancreatography (MRCP) confirmed these findings and showed a normal biliary tree and pancreas (Figure 2).

**Figure 3:**
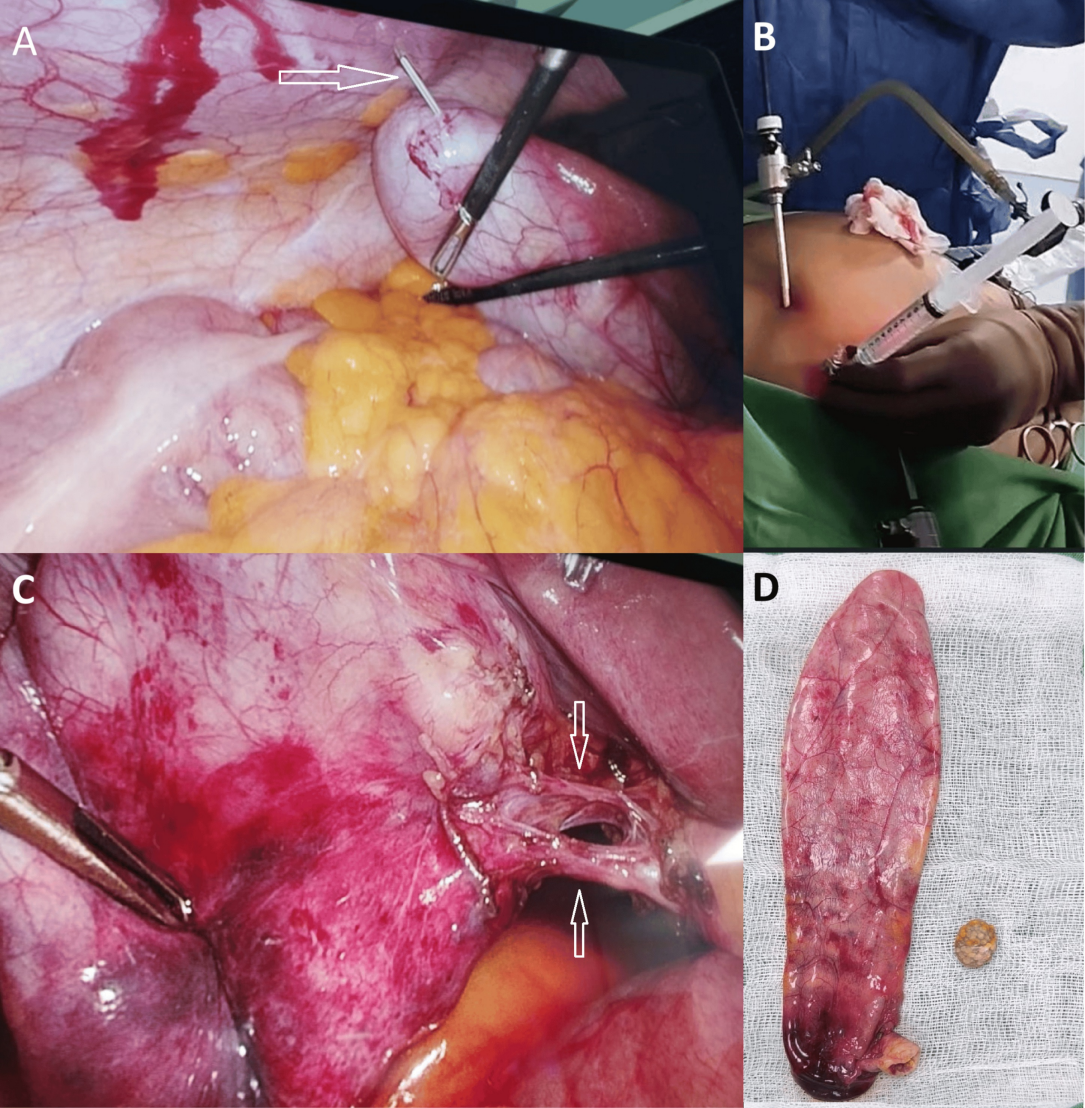
Intraoperative finding during cholecystectomy A: Percutaneous aspiration of distended gallbladder (white arrow), B: Aspirated clear fluid in syringe, C: Cystic duct (lower arrow) and cystic artery (upper arrow), D: the gallbladder and the impacted stone.

The patient underwent a laparoscopic cholecystectomy, which revealed a distended, elongated gallbladder that was difficult to grasp and was assigned a Parkland Grading Scale grade of 3 (Figure 3A). We therefore performed percutaneous decompression using a 16-gauge cannula, aspirating approximately 125 mL of clear fluid (Figure 3A, 3B). The remainder of the procedure was uneventful. The patient was discharged on postoperative day 1 and remained asymptomatic at her two-week follow-up appointment. Histopathology came back as chronic cholecystitis. The gallbladder was 145 mm long, and the wall thickness was 2 mm.

## Discussion

Gallbladder hydrops in humans is underreported in the literature and lacks large-scale studies to deeply analyze its risk factors and potential complications. Most of the available information comes from animal studies [11,12]. Murtaza Khomusi et al. reported one case (0.4%) among the cases they collected over two years. Khan et al., in their 26-year experience, encountered 216 mucoceles, constituting 4.2% of the total number of cholecystectomies performed. Similarly, Shirah et al. reported 57 cases (5.8%) over 10 years [12-14]. Gallbladder hydrops is a condition that most surgeons will encounter in their careers. That said, its definition is loosely applied. In this literature review, seven of the 14 case reports involving gallbladder removal did not describe the filling fluid and focused only on size (Table 2) [1,7-10,15,16]. The remaining reports described the fluid as clear, white, or purulent mucus or mucoid material (Table 2).

**Table 2:** Key characteristics of the case reports included in this literature review MRCP: magnetic resonance cholangiopancreatography

Case report (Author, Year)	Age/Gender	Presenting symptoms	Duration of symptoms	First diagnostic image	Stone size	Gallbladder length	Fluid description	Treatment received
Tan et al., 2025 [1]	18/Female	Acute cholecystitis	2 days	CT	Not mentioned	145 mm	Not mentioned	Laparoscopic cholecystectomy
Haji et al., 2023 [2]	28/Male	Gastric outlet obstruction	2 years	Ultrasound	No stone	149 mm	Purulent mucoid bile	Open subtotal cholecystectomy
Sharma et al., 2021 [3]	50/Female	Right hypochondrial pain	3 days	CT	Not mentioned	Not mentioned	-	Not mentioned
Le et al., 2023 [4]	56/Female	Abdominal discomfort and nausea	4 days	Ultrasound	Not mentioned	217 mm	Clear mucous-like fluid	Laparoscopic cholecystectomy
Viswanathan and Cheng, 2012 [5]	84/Female	Abdominal discomfort, weight loss, and jaundice	No mentioned	Ultrasound	No stones	120 mm	-	Conservative
Viswanathan et al., 2012 [5]	83/Male	Asymptomatic	-	MRCP	Not mentioned	Not mentioned	-	Conservative
Loh et al. 2019 [6]	63/Male	Gastric outlet obstruction	6 months	CT	10 mm	98 mm	White mucoid bile	Open cholecystectomy
Adhikari et al., 2024 [7]	79/Male	Dark urine and pale stool	1 week	CT	No stone	102 mm	Not mentioned	Laparoscopic cholecystectomy
Amarnath et al., 2019 [8]	78/Male	Epigastric pain	1 day	CT	No stone	110 mm	Not mentioned	Laparoscopic subtotal cholecystectomy
Reyes et al., 2017 [9]	39/Female	Acute cholecystitis	4 days	Ultrasound	6.9 mm	139 mm	Not mentioned	Laparoscopic cholecystectomy
Yadav and Kankaria, 2017 [10]	46/Female	Right hypochondrial pain	Not mentioned	Ultrasound	10.6 mm	300 mm	Not mentioned	Laparoscopic cholecystectomy
Poget et al., 2024 [11]	51/Female	Right hypochondrial pain	6 months	Ultrasound	6 mm	110 mm	Whitish mucus	Laparoscopic cholecystectomy
Ganta et al., 2022 [15]	67/Female	Acute cholecystitis	4 days	Ultrasound	Not mentioned	170 mm	Not mentioned	Laparoscopic cholecystectomy
Mvoula et al., 2023 [16]	42/Female	Acute cholecystitis	7 days	Ultrasound	15 mm	155 mm	Not mentioned	Laparoscopic cholecystectomy
Ercihan et al., 2022 [17]	26/Female	Upper abdominal pain and indigestion	2 years	Ultrasound	14 mm	75 mm	Mucoid material	Laparoscopic cholecystectomy
Ercihan et al., 2022 [17]	34/Female	Upper abdominal pain and nausea	1 months	Not done	No stones	70 mm	Mucus	Laparoscopic cholecystectomy
Valecha et al., 2019 [18]	27/Female	Abdominal pain, vomiting	3 days	Ultrasound	Not mentioned	85 mm	Whitish material	Laparoscopic cholecystectomy
Our case	63/Female	Intermittent right hypochondrial pain	4 years	Ultrasound	13 mm	145 mm	Clear serous fluid	Laparoscopic cholecystectomy

The majority of case reports use "gallbladder hydrops" and "gallbladder mucocele" interchangeably. However, a more nuanced distinction is highlighted in several reports. They posit that a hydropic gallbladder results from chronic cystic duct obstruction, leading to the accumulation of a watery, serous fluid. A mucocele, in contrast, is defined by mucinous hyperplasia of the gallbladder epithelium and an overproduction of mucus, which is also accompanied by obstruction [11]. Furthermore, some cases involve mucinous metaplasia without physical obstruction, while hydrops can develop in the absence of stones, potentially due to a functional obstruction of the gallbladder outlet [2,7,8,11]. All these factors should be considered when describing gallbladder hydrops.

Our case involves a 63-year-old woman with a long-standing hydropic gallbladder, which was intermittently symptomatic, radiologically confirmed, and ultimately treated with laparoscopic cholecystectomy and intraoperative decompression. The gallbladder was distended with clear watery fluid due to a stone impacted in the neck. The absence of mucinous hyperplasia on histopathology confirms the diagnosis of hydrops rather than mucocele. The chronic presentation, diagnostic definition, and successful surgical outcome provide several reference points for comparison with similar cases in the literature.

The most common cause of gallbladder hydrops is cholelithiasis, as seen in this case. Other possible causes of gallbladder outflow obstruction include benign or malignant polyps, cystic duct fibrosis, external compression from either liver parenchyma or perihilar lymphadenopathy, intraluminal parasites, or functional disorders [2,7].

Hydropic gallbladder has been reported in all age groups (though the pediatric population was not included in this literature review) and is more common in females (Table 2). Its presentation ranges from an incidental finding to chronic pain to acute conditions such as acute cholecystitis [5,7,11,17]. Our patient presented with chronic intermittent pain but no systemic inflammatory symptoms and had normal liver function tests. Older patients tend to present with chronic symptoms or may even remain asymptomatic, and may have had the condition for many years before diagnosis [4]. Interestingly, some patients develop atypical manifestations such as right lower quadrant pain or urinary symptoms, resulting from mechanical compression of nearby structures by the hydropic gallbladder [1,4]. Our patient also initially presented with urinary symptoms, similar to the case reported by Le et al. [4].

Although some cases of gallbladder hydrops may remain asymptomatic for long periods, particularly in elderly patients, most cases present with symptoms, and some may even develop complications. In our patient, the gallbladder size did not increase significantly despite three years of observation, though she experienced intermittent pain or discomfort. Potential complications primarily arise from the compression of adjacent structures, leading to Mirizzi syndrome or gastric outlet obstruction. Additionally, empyema may develop, and in rare cases, perforation can occur [2,6-8]. Although these complications are considered uncommon - as evidenced by Shirah et al.'s series of 57 cases, over 10 years, without any - our review of 17 case reports revealed two instances of gastric outlet obstruction, one of Mirizzi syndrome, and one perforation [12].

This higher rate is likely skewed, as case reports have an inherent bias toward publishing complicated presentations. Another potential complication is malignant transformation. This is more likely with a mucocele due to the associated epithelial hyperplasia and metaplasia, which carry a risk of dysplasia and can lead to carcinoma. The incidence of gallbladder cancer in cholecystectomy specimens has been reported to be around 1.5%. Additionally, the presence of the mucocele or the gallbladder hydrops can be secondary to gallbladder neoplasm [11].

The longest reported gallbladder measured 300 mm [10]. In our literature review, gallbladder size ranged from 70 to 300 mm, with a median of 115 mm (Table 2). White mucus was observed even in gallbladders of normal size (70-100 mm) [2,17]. Only six case reports specified the size of the impacted stone, which ranged from 6 mm to 15 mm [6,9-11,16,17], while five case reports documented no stones [2,5,7,8,17].

Most cases of gallbladder hydrops can be diagnosed via abdominal ultrasound. However, a CT scan or MRCP is often performed to better characterize the condition, rule out other pathologies, or when ultrasound findings are limited (e.g., due to obesity or bowel gas) [9]. In five cases, CT scans were the initial imaging study because the diagnosis was unclear, and the hydropic gallbladder was not suspected and discovered after imaging (Table 2). In our patient, gallbladder hydrops was initially detected on ultrasound. Nevertheless, an abdominal CT was obtained during the first presentation, while an MRCP was obtained during the second presentation. These additional imaging modalities allowed for further assessment of gallbladder size and confirmed the absence of bile duct stones or anatomical abnormalities.

The standard treatment for gallbladder hydrops is cholecystectomy. This approach is justified by the frequent presence of symptoms, the risk of complications (including gallbladder rupture), and the potential for malignant transformation in longstanding mucoceles due to epithelial metaplasia [11]. Conservative management may be considered in asymptomatic elderly patients when the risks of surgery outweigh the potential complications, but the long-term outcome has not been studied, and the possible complications should be discussed carefully with the patient [5]. In the reported cases (Table 2), cholecystectomy was performed in all but three instances: one patient was referred for surgery, while the other two were elderly with minimal symptoms and were managed conservatively [3,5].

Although gallbladder hydrops can complicate laparoscopic cholecystectomy, the procedure was successfully completed in most cases (Table 2). Two cases required conversion to open cholecystectomy - one due to encapsulated peritonitis and the other due to dense adhesions [2,6]. Laparoscopic cholecystectomy was successfully performed by Shirah et al. in all 57 of their reported cases [12]. Percutaneous cholecystostomy serves as a temporary measure to decompress the gallbladder, alleviate symptoms, and stabilize patients, either as a bridge to surgery or in high-risk individuals. Endoscopic ultrasound-guided drainage is an alternative minimally invasive option [12].

One of the challenges in operating on gallbladder hydrops is the distended gallbladder, which is difficult to manipulate and carries a risk of rupture; therefore, Intraoperative decompression - via laparoscopic needle aspiration or percutaneous cannula - is often needed but risks bile spillage. To mitigate this, some surgeons place a figure-of-eight suture around the puncture site for immediate closure after aspiration. Notably, only four reported cases mention gallbladder decompression, none of which specify the technique used [2,4,6,15]. However, Shirah et al. performed percutaneous drainage for all reported cases without complications and recommended it to ensure safe laparoscopic cholecystectomy in gallbladder hydrops [12]. We aspirated the gallbladder contents via a 16-gauge cannula introduced through the abdominal wall into the fundus of the gallbladder under laparoscopic guidance. After decompression, the puncture site was secured with a gallbladder grasper to minimize spillage.

## Conclusions

Gallbladder hydrops, primarily caused by cystic duct obstruction from an impacted stone, leads to the absorption of bile and accumulation of sterile "white bile." While well-documented in animals, it is underreported in humans and presents a diagnostic challenge due to its highly variable presentation, from being asymptomatic to causing chronic pain or atypical compressive symptoms. Abdominal ultrasound is the key initial diagnostic tool, though CT or MRCP are often essential for confirmation, detailed anatomical mapping, and excluding other pathologies or malignancies before surgical intervention.

Laparoscopic cholecystectomy remains the definitive treatment for symptomatic cases. However, the surgery is technically challenging due to the large, tense gallbladder, which increases the risk of intraoperative perforation. Therefore, strategic intraoperative decompression is a crucial and often necessary step to safely collapse the organ and facilitate dissection. A successful outcome hinges on preoperative recognition of this condition and meticulous surgical planning.

## References

[1] Tan S, Adams Z, Rudkin S, Matonis D (2025). A case report of hydropic gallbladder presenting as right lower quadrant abdominal pain. J Educ Teach Emerg Med.

[2] Haji AG, John AR, Elhassan A, Zohdy M, Haider SI, Lourdusamy S, Piramanayagam B (2023). A rare case of ‘gastric outlet obstruction secondary to hydrops of gallbladder’ caused by recurrent acalculous cholecystitis: a variant of Bouveret’s syndrome - a rare case and review of literature. Surg Gastroenterol Oncol.

[3] Sharma R, Stead TS, Aleksandrovskiy I, Amatea J, Ganti L (2021). Gallbladder hydrops. Cureus.

[4] Le ZH, Dowling L, Ranasinghe SM (2023). A rare case of calculous gallbladder hydrops presenting with atypical abdominal and urinary symptoms. Cureus.

[5] Viswanathan P, Cheng E (2012). Management of hydrops gallbladder in the elderly: 806. Off J Am Coll Gastroenterol ACG.

[6] Loh WL, Ng NZ, Kabir T, Chan CY (2019). Rare case of gallbladder mucocele causing gastric outlet obstruction treated with cholecystectomy. Int J Surg Case Rep.

[7] Adhikari B, Nieto LM, Adhikari B, Dhital A, Attar C (2024). Hydrops gallbladder caused by cystic duct fibrosis leading to Mirizzi syndrome: a case report. Cureus.

[8] Amarnath S, Polavarapu AD, Gumaste V (2019). Spontaneous perforation of an acalculous hydropic gallbladder in a diabetic patient with neuropathy: an underdiagnosed entity. Gastroenterology Res.

[9] Reyes Q, McLeod RL, Fernandes K, Muralidharan V, Weinberg L (2017). Magnetic resonance cholangiopancreatography uncovering massive gallbladder mucocele in a patient with ambiguous clinical and laboratory findings: A case report. Int J Surg Case Rep.

[10] Yadav R, Kankaria J (2017). Longest gallbladder: a case report. Int J Surg Case Rep.

[11] Poget M, Salvatori Chappuis V, Carbó Descamps F, Saadi A (2024). Gallbladder mucocele caused by intestinal metaplasia in lithiasic cholecystitis: a case report and literature review of a rare association. Int J Surg Case Rep.

[12] Shirah BH, Shirah HA, Albeladi KB (2018). The value of intraoperative percutaneous aspiration of the mucocele of the gallbladder for safe laparoscopic management. Updates Surg.

[13] Murtaza Khomusi M, Parveen S, Iqbal M (2022). Prevalence of empyema or mucocele or other histological diagnoses in patients undergoing cholecystectomy with diagnosis of chronic cholecystitis. Cureus.

[14] Khan KS, Sajid MA, McMahon RK, Mahmud S, Nassar AH (2020). Hartmann's pouch stones and laparoscopic cholecystectomy: the challenges and the solutions. JSLS.

[15] Ganta N, Alnabwani D, Shah V, Imtiaz A, Bommu VJ, Cheriyath P (2022). A rare case of acute calculous cholecystitis with gallbladder hydrops. Cureus.

[16] Mvoula L, Khrisat T, Melton S (2023). A severely dilated gallbladder with multiple gallstones after concomitant laparoscopic sleeve gastrectomy and childbirth in a Hispanic woman. Cureus.

[17] Ercihan E, Küçük Ş, Arslan RS, Küçük İG (2022). Gallbladder mucocele: two case reports. MEVLANA Med Sci.

[18] Valecha B (2019). A case of mucocele gallbladder with review of literature. Saudi J Pathol Microbiol.

